# Biochemical and Ultrasonographic Parameters Predicting Long-Term Hypothyroidism After Subacute Thyroiditis

**DOI:** 10.3390/ijms26094176

**Published:** 2025-04-28

**Authors:** Andrea Corsello, Piergiacomo Maria Cacciamani Fanelli, Luisa Lener, Gianluca Cera, Pietro Locantore, Alfredo Pontecorvi, Salvatore Maria Corsello, Rosa Maria Paragliola

**Affiliations:** 1 Unit of Endocrine Surgery, Ospedale Isola Tiberina-Gemelli Isola, 00186 Rome, Italy; andrea.corsello@fbf-isola.it; 2 Unit of Endocrinology, Department of Translational Medicine and Surgery, Università Cattolica del Sacro Cuore, Fondazione Policlinico “A. Gemelli” IRCCS, 00136 Rome, Italy; piergiacomo.cacciamani01@icatt.it (P.M.C.F.); luisa.lener@gmail.com (L.L.); gianluca.cera@outlook.com (G.C.); pietro.locantore@icloud.com (P.L.); alfredo.pontecorvi@unicatt.it (A.P.); salvatoremaria.corsello@unicamillus.org (S.M.C.); 3 Departmental Faculty of Medicine, Unicamillus-Saint Camillus International University of Health Sciences, 00131 Rome, Italy

**Keywords:** subacute thyroiditis, long-term hypothyroidism, predictive factors

## Abstract

Subacute thyroiditis (SAT) represents an inflammatory disease of the thyroid gland, often resulting from viral infections or post-viral inflammatory responses. Long-term hypothyroidism is a possible evolution, requiring frequent follow-up and, if necessary, levothyroxine (LT4) replacement therapy. We retrospectively included 139 patients (out of 428) with SAT referring to the “Fondazione Policlinico Universitario A. Gemelli IRCCS” (Rome), between 2010 and 2022 to identify predictive parameters for long-term hypothyroidism. We evaluated TSH, FT4, and FT3 at four timepoints (diagnosis, 4–8 weeks, 10–20 weeks, and 28–54 weeks). We started LT4 therapy in patients with TSH > 10 μUI/mL or between 4–10 μUI/mL, with symptoms of hypothyroidism. “Long-term hypothyroidism” was defined as TSH > 4 μUI/mL after LT4 reduction and/or withdrawal. Univariate analysis showed correlations between long-term hypothyroidism and higher FT3 and FT4 and positive anti-Tg Abs at diagnosis and higher changes in TSH values (ΔTSH), decreased thyroid volume, and persistence of hypoechoic areas during follow-up. Furthermore, more severe thyrotoxicosis at presentation may be associated with a faster progression to hypothyroidism, likely due to greater thyroid damage. Multivariable analysis found ΔTSH (TP2-TP1) as an independent predictor of hypothyroidism. We propose specific biochemical and ultrasonographic parameters at diagnosis and during follow-up as possible predictors of long-term hypothyroidism after SAT, reducing treatment and healthcare costs for most patients who will never require replacement therapy.

## 1. Introduction

Subacute thyroiditis (SAT) is an inflammatory disease of the thyroid gland caused by a viral infection or a post-viral inflammatory process [1]. Both mechanisms trigger a predominantly cell-mediated response. Although direct evidence is limited, several indirect observations support this hypothesis: approximately 60% of patients report symptoms of a viral infection, particularly of the upper respiratory tract, 2–8 weeks prior to the onset of SAT-specific symptoms. Additionally, immunoglobulins targeting various viral antigens can be detected in the patient’s serum, and the disease often occurs in seasonal clusters, mirroring the patterns of certain viral infections. Several viral agents, including influenza virus, mumps virus, measles virus, adenoviruses, Epstein-Barr virus, and hepatitis E virus, have been implicated as potential triggers for SAT [1,2,3,4]. SARS-CoV-2, the virus responsible for the COVID-19 pandemic spread, has also been associated with SAT [5], recently identified as the most frequent COVID-19-related thyroiditis [6]. A plausible molecular mechanism involves the high expression of angiotensin-converting enzyme 2 (ACE2) and transmembrane serine protease 2 (TMPRSS2) in thyroid cells. These proteins form the primary molecular complex utilized by SARS-CoV-2 to enter host cells, similar to its mechanism in the respiratory tract [6]. Moreover, SAT has been linked to COVID-19 vaccination [7]. A hypothesized etiopathogenetic mechanism suggests that viral infection, along with genetic or autoimmune predisposition, could trigger molecular mimicry between the spike protein of the virus and antigens expressed by thyroid follicular cells [8]. However, there is no evidence that a specific viral agent influences the clinical course of the disease once the inflammatory process is initiated [3]. Instead, data in the literature shows an association between SAT and certain HLA class I and II alleles: HLAB35 (more than 60% of patients with SAT are positive for this antigen [9]), HLA-B67, HLA-DRB1:01, HLA-C04:01 and HLA-B18:01 (the latter seems to be linked to a higher risk of recurrence [10]).

Unlike chronic autoimmune thyroiditis, where the antigen presence sustains ongoing inflammation, the antigen on SAT is transient, resulting in a self-limiting disease, characterized by a multiphasic course of thyroid dysfunction. This course typically begins with an initial thyrotoxic phase, which occurs due to follicular damage and usually lasts 3–6 weeks. This is followed by a transient hypothyroid phase, which can last up to 6 months, after which patients generally recover to a state of euthyroidism [11]. Importantly, only a minority of patients (5–15%) will progress to develop long-term or permanent hypothyroidism [11]. The clinical course of SAT, thus, includes a fluctuating pattern of thyroid dysfunction, which poses challenges in predicting the long-term outcomes of the disease. To ensure optimal patient care, it is crucial to closely monitor patients during this multiphasic progression. For those who develop persistent hypothyroidism, a more intensive clinical, biochemical, and imaging follow-up is necessary to determine the optimal timing for initiating levothyroxine (LT4) replacement therapy. Despite its importance, only a limited number of studies have investigated the potential predictors of progression to permanent hypothyroidism [12]. The primary focus of the present study is to explore and evaluate the variables that may predict the development of long-term hypothyroidism following SAT. Specifically, the study aims to identify clinical, biochemical, and instrumental parameters observed at disease onset, as well as throughout the follow-up period, which could help in forecasting patient outcomes. In addition, the research will consider treatment-related factors, such as the use of corticosteroids, non-steroidal anti-inflammatory drugs (NSAIDs), or a combination of both. Understanding these factors will enable better prediction models and provide essential insights into how to approach treatment strategies and follow-up protocols. By identifying predictors of permanent hypothyroidism, this research may support personalized care and reduce unnecessary follow-up in low-risk patients, leading to more efficient use of healthcare resources.

## 2. Results

**Figure 1:**
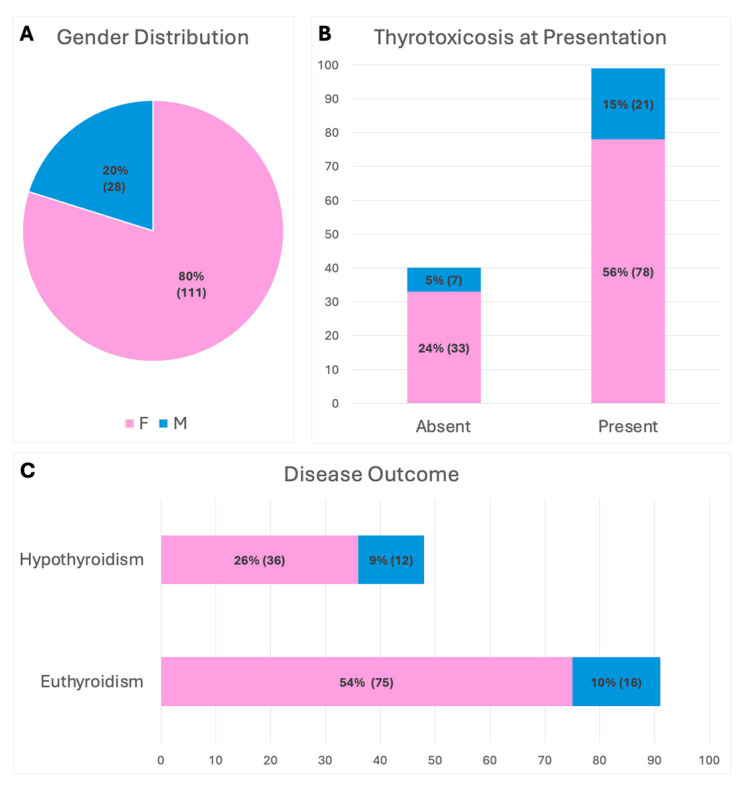
(**A**) Gender distribution of the cohort. (**B**) Laboratory evidence of thyrotoxicosis at disease presentation. (**C**) Disease outcome.

The studied cohort of 139 patients, (111 females and 28 males; F:M ≈ 4:1) had a mean age of 48.5 years. (Figure 1A).

At the time of diagnosis, 99 patients (71%) showed laboratory evidence of thyrotoxicosis (TSH < 0.4 μUI/mL) (Figure 1B).

During follow-up, only a small number of patients who were euthyroid at baseline (TP0) developed thyrotoxicosis (4 patients at TP1).

In our cohort, 48 patients (35%) developed long-term hypothyroidism (hypothyroid group) at the last follow-up and 91 (65%) had normal thyroid function (Figure 1C).

### 2.1. Univariate Analysis of Variables at Disease Presentation

#### 2.1.1. Clinical Variables and Inflammatory Index

No relationship has been observed between clinical variables, biochemical indexes of inflammation, and disease progression (Supplementary Table S1).

#### 2.1.2. Thyroid Function

**Figure 2:**
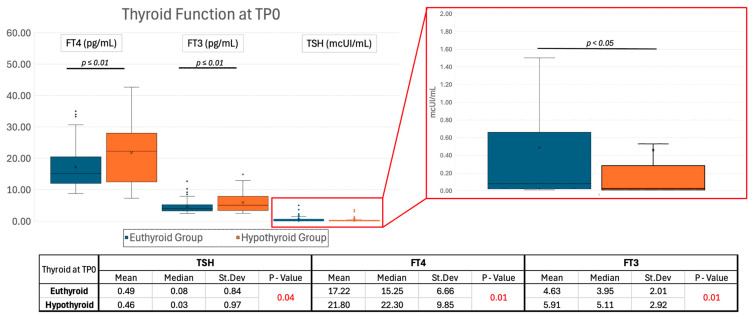
Descriptive and inferential statistics of thyroid function parameters at disease presentation. Box and Whisker Plot of thyroid function tests at diagnosis. The central line within each box represents the median, the central × the mean, the edges of the box indicate the interquartile range (IQR), and the whiskers extend to the smallest and largest values within 1.5 times the IQR from the quartiles. Outliers are depicted as individual points. Abbreviations: FT4, free thyroxine; FT3, free triiodothyronine; TSH, thyroid-stimulating hormone; St.Dev., standard deviation.

As reported in Figure 2, mean TSH at TP0 between the euthyroid and hypothyroid groups showed a small and marginally significant (*p* = 0.04) difference. In contrast, the FT4 values at TP0 showed a pronounced and significant (*p* = 0.01) difference between groups. Additionally, the euthyroid group showed a significantly lower mean FT3 at diagnosis compared to the hypothyroid group (*p* = 0.01).

#### 2.1.3. Disease Severity Stratification

At disease presentation, patients were stratified by thyrotoxicosis severity and categorized into mild, moderate, and severe groups.

**Figure 3:**
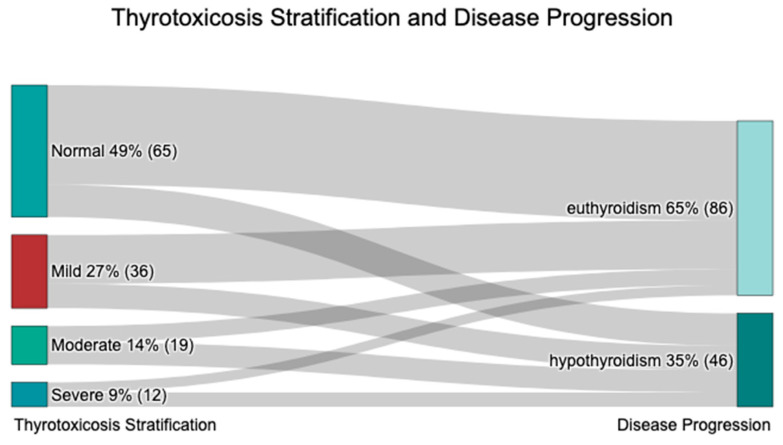
Thyrotoxicosis severity and disease progression.

The euthyroid group had a significantly lower thyrotoxicosis severity compared to the hypothyroid one (*p* = 0.003, Table 1). A euthyroid outcome was observed 51% more frequently in patients with normal FT4 levels than in those who progressed to hypothyroidism (75.38% vs. 24.62%, Table 1, Figure 3). Similarly, mild thyrotoxicosis included 33% more euthyroid patients than hypothyroid patients (66.67% vs. 33.33%). In contrast, moderate thyrotoxicosis was associated with a 15% higher frequency of long-term hypothyroidism (57.89% vs. 42.11%), while severe thyrotoxicosis was linked to a 17% higher frequency of hypothyroidism (58.33% vs. 41.67%).

**Figure 4:**
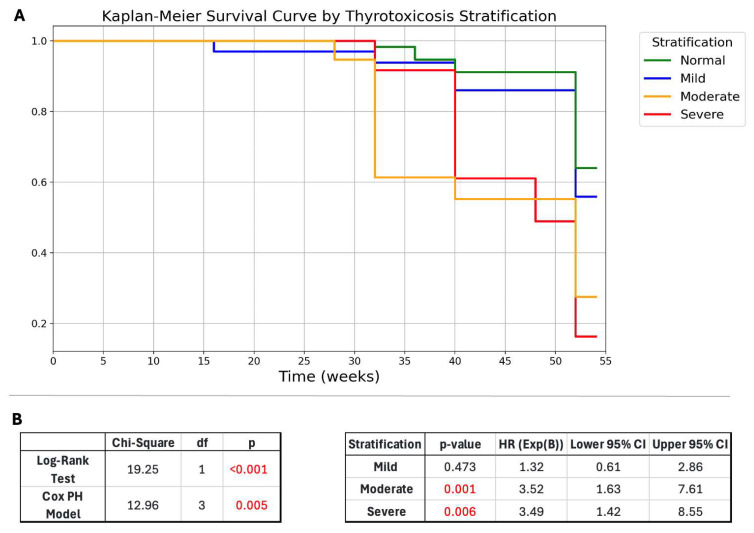
(**A**) Kaplan-Meier survival curves for hypothyroidism onset stratified by thyrotoxicosis severity at presentation. Patients with severe and moderate thyrotoxicosis experienced an earlier onset of hypothyroidism, whereas those with mild thyrotoxicosis or normal FT4 levels had a delayed progression. (**B**) The log-rank test confirmed a statistically significant difference in the survival curves. The Cox-PH model resulted significantly. Abbreviations: HR, hazard ratio; CI, confidence interval; PH, proportional hazard; df, degrees of freedom; Exp (**B**), exponentiated coefficient; HR, hazard ratio.

A more severe thyrotoxicosis at presentation may be associated with a faster progression to hypothyroidism, likely due to greater thyroid damage. To assess the impact of thyrotoxicosis severity on disease progression, a Kaplan-Meier survival analysis and log-rank test were performed based on thyrotoxicosis stratification at presentation (Figure 4A,B). The survival curves illustrate a differential time-to-event distribution, with patients in the severe and moderate thyrotoxicosis groups developing hypothyroidism earlier, whereas those with mild thyrotoxicosis or normal FT4 levels remained euthyroid for a longer duration (Figure 4A). The log-rank test confirmed a statistically significant difference in survival curves (*p*-value < 0.001; Figure 4B), supporting the hypothesis that higher thyrotoxicosis severity accelerates hypothyroidism onset.

The Cox proportional hazards model was statistically significant overall (*p* = 0.005, Figure 4B) in predicting the risk of hypothyroidism. Compared to the normal thyrotoxicosis group, the moderate (HR = 3.49, *p* = 0.006, Figure 4B) and severe (HR = 3.52, *p* = 0.001, Figure 4B) groups had a significantly higher hazard of developing hypothyroidism, while the mild group (HR = 1.32, *p* = 0.473, Figure 4B) showed no significant difference.

Conclusively, thyrotoxicosis stratification severity is a significant predictor of disease progression and timing of hypothyroidism onset.

#### 2.1.4. Autoantibodies

**Figure 5:**
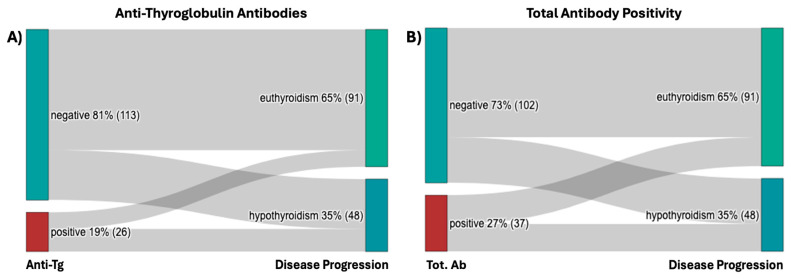
(**A**) Anti-Tg Abs results at presentation and disease progression; (**B**) Total antibody positivity results at presentation and disease progression. Abbreviations: Anti-Tg, anti-thyroglobulin; Tot. Ab, total antibody.

In our cohort, about 80% of patients tested negative for anti-Tg Abs (Figure 5A). Anti-Tg Abs positive patients showed approximately a 30% higher proportion of progression to hypothyroidism (58% vs. 29%, *p* = 0.006) (Table 2, Figure 5A). Anti-TPO Abs positivity showed no statistical significance.

When considered together, anti-Tg Abs positive patients or anti-TPO Abs positive patients showed a 20% increased proportion of progression to hypothyroidism (respectively 50% vs. 30%, *p* = 0.035) (Table 2, Figure 5B).

The patients were stratified into three groups according to thyroid dimensions and compared with respect to antibody titers. This analysis was performed to evaluate the potential coexistence of autoimmune thyroiditis—either primary or SAT-induced—with SAT. Autoimmune thyroiditis is typically associated with an initial increase in thyroid size, followed by a decrease in later stages [13].

In our cohort, only one patient (Table 3) exhibited a reduction in thyroid volume, possibly due to preexisting autoimmune thyroiditis. Most patients had normal or increased thyroid dimensions, likely attributable to SAT alone or in combination with SAT-induced autoimmune thyroiditis. Statistical analysis revealed no significant correlation between antibody titers and thyroid dimensions, suggesting that autoimmune thyroiditis was absent in our patients. Thus, thyroid size changes, if present, were driven solely by SAT, and differences in thyroid dimensions across groups were not influenced by antibody titers.

#### 2.1.5. Ultrasound (US)

**Figure 6:**
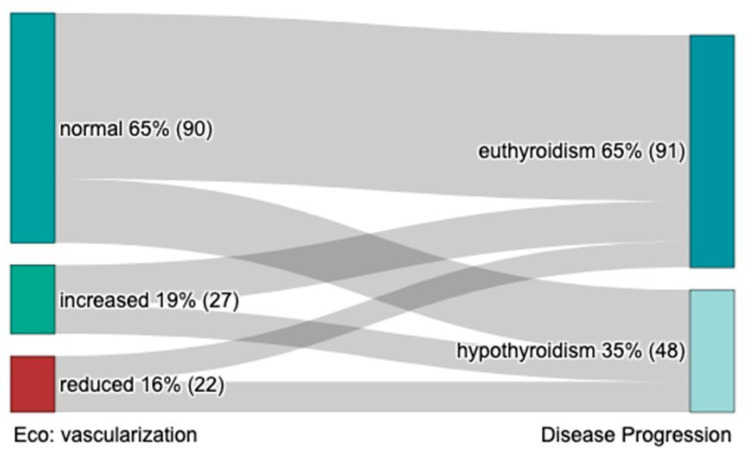
Vascularization and disease progression.

US characteristics at diagnosis are reported in Table 4. Thyroid size and the presence of thyroid nodules or pseudo-nodular areas at diagnosis were unable to predict the course of SAT. At univariate analysis, reduced thyroid vascularization at diagnosis (15% of patients) showed a 28% higher proportion of long-term hypothyroidism (55% vs. 27% respectively, *p* = 0.036), compared with normal vascularization (64% of patients) (Table 4, Figure 6). Increased vascularization, observed in 19% of patients, was associated with approximately a 14% higher rate of hypothyroidism compared to those with normal vascularization (41% vs. 27%, *p* = 0.036) (Table 4; Figure 6). 

#### 2.1.6. Therapy

About 50% of patients were prescribed NSAIDs, while 79% were prescribed GCSs. 37% of the patients used a combination of NSAIDs and GCSs, while only 8% did not use any anti-inflammatory medication. No significant association between treatment and thyroid function outcome was observed (Supplementary Table S2).

### 2.2. Univariate Analysis of Variables During Disease Follow-Up

Throughout the follow-up period, most patients were scheduled for checkups at TP1 (4 to 8 weeks) TP2 (10 to 20 weeks), and TP3 (28 to 54 weeks after diagnosis). At TP3, patients repeated a thyroid US.

#### 2.2.1. TSH Changes During Follow-Up

The distribution of TSH levels and corresponding descriptive statistics at TP0, TP1, TP2, and TP3 are presented in Supplementary Figure S1.

**Figure 7:**
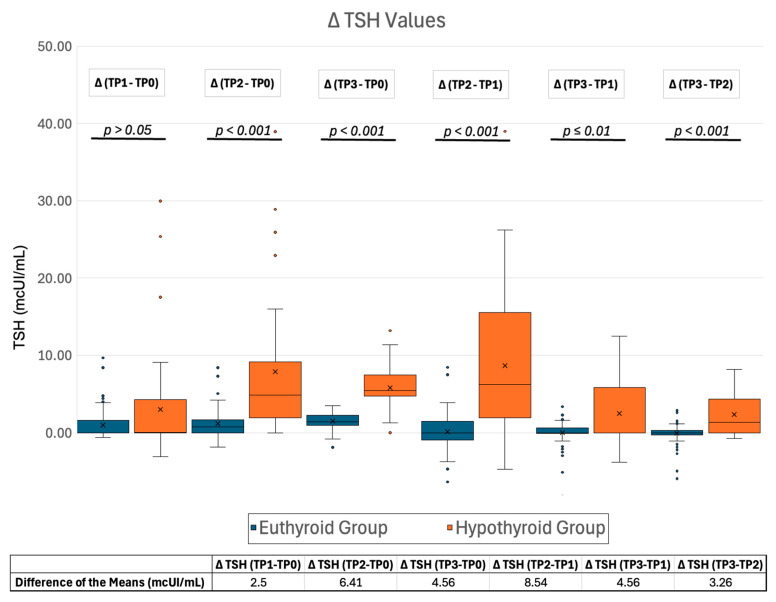
Box and Whisker plot of all ΔTSHs with relative *p*-values. Abbreviations: TSH, thyroid stimulating hormone; TP0, time point 0 (at disease presentation); TP1, time point 1 (first follow-up); TP2, time point 2 (second follow-up); TP3, time point 3 (third follow-up).

The change in TSH levels (ΔTSH) at different time points was calculated for each patient. ΔTSH values deviated from a normal distribution. Besides ΔTSH (TP1-TP0), all ΔTSHs showed considerable statistical significance in the progressive increment of TSH values between the euthyroid and hypothyroid groups (Figure 7, Supplementary Table S3). In particular, the magnitude of TSH change over time was significantly smaller in patients who were eventually euthyroid at last follow-up (euthyroid group) than those who developed long-term hypothyroidism (hypothyroid group). The most obvious differences in ΔTSHs between the two groups were observed between time points TP2-TP1 and TP2-TP0 (Figure 7).

#### 2.2.2. FT4 Time Progression

**Figure 8:**
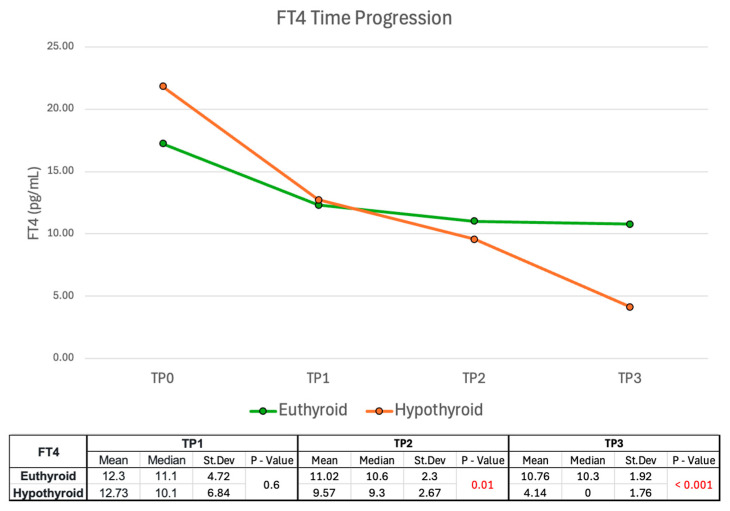
FT4 values during follow-up. FT4 values were approximated to zero at TP3 in patients undergoing LT4 treatment, as endogenous production was insufficient to meet physiological demands. Abbreviations: FT4, free thyroxine; St. Dev., standard deviation.

As reported in Figure 8, both groups exhibited a decline in FT4 mean levels during follow-up, but the decrease was faster in the hypothyroid group. Interestingly, at TP1 the FT4 values between the euthyroid and the hypothyroid groups were similar without significant differences, while at TP2 a notable shift occurred (*p* = 0.01). At TP3, the FT4 mean values between the two groups were similar (10.76 in the euthyroid group and 10.03 in the hypothyroid group). However, many patients in the hypothyroid group were receiving LT4 by this time. Consequently, LT4 values in treated patients were approximated to 0 as a mathematical approximation -acknowledging its limitations and insufficient endogenous hormone production. The Mann-Whitney U tests showed that FT4 values at TP2 and TP3 between the two functional groups were significant in predicting disease outcomes.

#### 2.2.3. FT3 Time Progression

**Figure 9:**
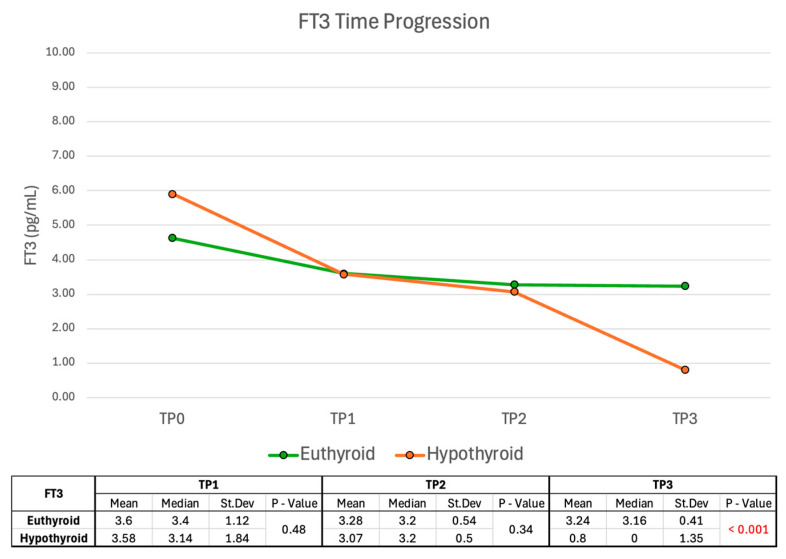
FT3 values during follow-up. FT3 values were approximated to zero at TP3 in patients undergoing LT4 treatment, as endogenous production was insufficient to meet physiological demands. Abbreviations: FT3, free triiodothyronine; St. Dev., standard deviation.

FT3 values show no significant difference between the euthyroid group and the hypothyroid group at TP1 or TP2, while FT3 levels were significantly higher in the euthyroid group at TP3 (*p* < 0.001) (Figure 9). However, similarly to FT4, the clinical and statistical significance of this finding at TP3 is low due to LT4 replacement therapy in some patients.

#### 2.2.4. LT4 Supplementation at TP3

LT4 supplementation at TP3 was administered to a minority of patients (27 cases). All patients who received LT4 ultimately progressed to long-term hypothyroidism, in contrast to the 18% who did not receive LT4 (*p* < 0.001) (Supplementary Table S4). Around 45% of patients who progressed to hypothyroidism did not necessitate LT4 supplementation at TP3. Therefore, a patient who does not require LT4 supplementation at TP3 still has a 20% chance of developing long-term hypothyroidism.

#### 2.2.5. Ultrasound (US)

US features at TP3 have been reported in Table 5. Notably, decreased thyroid volume was associated with a 36% higher proportion of hypothyroidism compared to normal thyroid volume (*p* = 0.003). A higher proportion of hypothyroidism was observed in patients with hypoechoic areas in the US at TP3 (*p* = 0.027). Specifically, while 20% of patients without hypoechoic areas progress to long-term hypothyroidism, this percentage increases to 40% among patients with hypoechoic areas.

Reduced echogenicity of the thyroid gland on ultrasound is a characteristic feature of inflammatory conditions, such as HT. Therefore, the persistence of a hypoechoic area may indicate an increased risk of long-term hypothyroidism due to underlying HT. As previously mentioned, we identified anti-Tg Abs as a factor associated with a higher proportion of long-term hypothyroidism, whereas positivity for anti-TPO Abs did not show statistical significance.

At TP3, there is no statistically significant relationship between thyroid vascularization or the presence of nodules and disease progression. A notable difference exists in the US findings between TP0 and TP3: there is an approximate 20% increase in the number of patients with no echogenic areas at TP3 when compared to TP0 (Table 5, “Resolution”). In 85% of patients who experienced apparent healing of the thyroid gland in the US, thyroid function was restored. Conversely, patients without healing of the thyroid gland had a 25% lower rate of euthyroidism achievement (*p* = 0.01) (Table 5).

### 2.3. Multivariable Analysis

A multivariable analysis was performed by evaluating key parameters at disease presentation and during follow-up. Specific clinical, instrumental, and biochemical parameters were selected for analysis based on both mathematical and clinical rationale to ensure the inclusion of the most representative variables. The refinement of predictor selection was further guided by mathematical considerations, taking into account potential influences such as model complexity, overfitting, sample size, and possible hidden suppression or confounding effects (Supplementary Table S5) [14,15]. Conclusively, the following predictors were chosen: (1) anti-Tg Abs; (2) US—Vascularization at TP0; (3) ΔTSH (TP2-TP1); (4) US—Dimensions at TP3.

Multivariable analysis (Table 6) showed that ΔTSH (TP2-TP1) is a significant predictor of hypothyroidism (OR 1.3, 95% CI 1.07–1.57, *p* = 0.007) together with Anti-Tg Ab positivity (OR 9.3, 95% CI 0.99–87.95, *p* = 0.051), even though this predictor has borderline significance. Thyroid vascularization patterns at TP0 and thyroid dimensions at TP3 were not significant predictors of hypothyroidism at multivariable analysis.

## 3. Discussion

In this study, we retrospectively analyzed a cohort of patients with subacute thyroiditis (SAT) and identified potential parameters that may predict the progression to long-term hypothyroidism. Previous studies have shown that early, transient hypothyroidism is common in patients with SAT, whereas permanent hypothyroidism is relatively rare, occurring in 5.9–15% of cases [16,17,18,19]. In our study, we observed a much higher incidence of long-term hypothyroidism (34%), which may reflect differences in diagnostic criteria between long-term and permanent hypothyroidism. Although gender was not identified as a significant factor influencing disease progression, a trend toward a higher risk of developing long-term hypothyroidism was observed in male patients in our cohort (43% of males vs. 31% of females, *p* = 0.44). One possible explanation is the reported anti-inflammatory effects of estrogens during viral infections, suggesting a potential protective role in SAT that cannot be excluded [20]. However, other authors have reported a significantly higher prevalence of SAT in females [21]. According to the clinical guidelines of the American Thyroid Association, nonsteroidal anti-inflammatory drugs (NSAIDs) are recommended for patients with mild pain, whereas glucocorticoids are preferred for those with moderate to severe symptoms [22]. The impact of treatment on the risk of developing hypothyroidism remains unclear. Görges et al. found no correlation between hypothyroidism and glucocorticoid use, although high cumulative doses of prednisolone were associated with a higher prevalence of hypothyroidism [21]. Conversely, a recent meta-analysis involving 1337 patients reported that the incidence of permanent hypothyroidism was lower in patients treated with glucocorticoids compared to NSAIDs. In our cohort, no significant difference was observed in the rate of developing hypothyroidism based on treatment (glucocorticoids vs. NSAIDs). This finding aligns with results published by Benbassat et al., who reported that patients treated with glucocorticoids experienced a shorter overall disease duration but no significant differences in the duration of hyperthyroidism, peak FT4 levels, or highest TSH levels compared to patients treated with NSAIDs [19].

Interestingly, stratifying patients into groups based on the severity of thyrotoxicosis at the time of diagnosis provides valuable insights into how the severity of the disease affects the likelihood and timing of progression to hypothyroidism. In our univariate analysis, FT3 and FT4 levels at diagnosis were significantly higher in the group of patients who developed long-term hypothyroidism. Kaplan-Meier analysis showed that severe thyrotoxicosis led to a faster progression to hypothyroidism, confirming a significant association (*p*-value < 0.001). This suggests that a more severe form of thyrotoxicosis at onset is more common among patients who fail to achieve euthyroidism following the resolution of inflammation. Furthermore, more severe thyrotoxicosis at presentation accelerates the onset of hypothyroidism, underlining the need for closer monitoring and intervention in these patients.

In a conventional paradigm, the severity of thyrotoxicosis directly correlates with the extent and duration of TSH suppression, leading to a slower recovery in more severe cases. This is particularly evident in Graves’ disease [23], where autoimmune mechanisms not only drive hormone overproduction but also likely prolong TSH suppression by modulating the hypothalamic-pituitary-thyroid axis, even after euthyroidism is restored. Conversely, in SAT, thyrotoxicosis results from the release of preformed thyroid hormones due to inflammation rather than increased synthesis. As a self-limiting, non-autoimmune condition, it does not chronically affect the thyroid axis, allowing for faster TSH recovery once inflammation resolves. Similar to postpartum thyroiditis, this can lead to a more rapid onset of hypothyroidism.

Interestingly, no correlation was found between long-term hypothyroidism and inflammatory markers. This finding aligns with a recent study involving 69 patients with SAT, which demonstrated that the Systemic Immune-Inflammation Index (SII) was associated with recovery time but did not show a significant correlation with long-term hypothyroidism [24]. TSH levels at diagnosis were not useful for assessing the degree of thyrotoxicosis. Although a significant difference was observed, the disparity between the two groups was small, limiting its clinical applicability. In contrast, our analysis revealed a more pronounced and significant difference in ΔTSH levels between the disease-progression groups. Specifically, the ΔTSH change was consistently smaller in the euthyroid group, suggesting that patients who reach euthyroidism maintain partial thyroid function during disease progression.

The most notable differences in ΔTSH between the two progression groups were observed at the TP2-TP1 and TP2-TP0 time points. These marked differences, coupled with their statistical significance, suggest that ΔTSH (TP2-TP0) and ΔTSH (TP2-TP1) could be effectively used in a clinical setting to predict disease progression. Notably, ΔTSH (TP2-TP1) was identified as an independent predictor of long-term hypothyroidism, with a 30% increased likelihood of developing hypothyroidism for each additional unit increase in TSH between TP2 and TP1 (OR = 1.3).

However, it is important to note that the ΔTSH values calculated using TSH at TP3 may have lower clinical relevance, as about 19% of patients received LT4 treatment between TP2 and TP3. FT4 levels, as expected, decreased in both groups in line with the progressive resolution of initial thyrotoxicosis. This decrease was more pronounced in the hypothyroid group, with the most significant change occurring at TP2. While the differences in FT4 levels at TP2 and TP3 between the two functional groups were statistically significant in predicting disease outcomes, these variations were less pronounced than those observed for TSH. Therefore, in clinical practice, TSH may serve as a more practical and reliable prognostic indicator than FT4 during SAT follow-up.

Regarding ultrasound findings, a reduced thyroid volume and the persistence of hypoechoic areas—potential markers of ongoing thyroid inflammation—were strong predictors of hypothyroidism risk in univariate analysis, consistent with previously published data [12]. Furthermore, our sub-analysis found no significant correlation between antibody titers and thyroid dimensions thyroid size changes were attributed solely to SAT.

Interestingly, our study found that patients with positive anti-Tg antibodies are at a higher risk of developing long-term hypothyroidism, whereas no significant association was observed for anti-TPO antibodies. Additionally, a higher prevalence of anti-Tg antibodies has been reported in SAT compared to anti-TPO antibodies [25]. The epitopes recognized by these autoantibodies in various thyroglobulin regions appear to differ between SAT and Hashimoto’s thyroiditis (HT). This suggests that the autoantibodies produced during acute inflammatory destruction in SAT are derived from a different molecular mechanism than those seen in “classical” autoimmune thyroid diseases [26].

We acknowledge that the retrospective design of our study may represent a limitation. Additionally, the study spans a long period, both before and after the onset of the SARS-CoV-2 pandemic. It is possible that COVID-19 infection has influenced the occurrence, frequency, and severity of SAT [27]. Despite these limitations, our findings suggest that FT3 and FT4 levels at diagnosis, TSH level variations at specific follow-up time points, the presence of anti-Tg antibodies, reduced thyroid volume, and the persistence of hypoechoic areas during follow-up may serve as potential predictors of long-term hypothyroidism following SAT. These parameters are more strongly associated with severe inflammation than traditional inflammatory markers. This approach holds promise for advancing personalized medicine in SAT management by enabling more tailored follow-up strategies and therapies for patients at higher risk of developing long-term hypothyroidism. At the same time, it may help reduce treatment and healthcare costs for the majority of patients who will not require replacement therapy.

## 4. Materials and Methods

### 4.1. Study Cohort and Evaluation Criteria

The study was approved by the Institutional Review Board. We retrospectively analyzed 428 patients who presented to the Endocrinology Unit of the “Fondazione Policlinico Universitario A. Gemelli IRCCS” (Rome) between January 2010 and January 2022 with a recent diagnosis of subacute thyroiditis (SAT).

The diagnosis of SAT was established based on a combination of clinical, biochemical, and imaging criteria. Patients were included if they presented with a typical clinical picture characterized by the acute onset of anterior neck pain, often radiating to the jaw or ears, associated with thyroid tenderness on palpation, low-grade fever, and symptoms of thyrotoxicosis such as palpitations, weight loss, heat intolerance, or tremor. Laboratory findings consistent with SAT included thyrotoxicosis, increased inflammatory markers (C-reactive protein and/or erythrocyte sedimentation rate), and elevated serum thyroglobulin. Imaging studies further supported the diagnosis, with thyroid ultrasound typically revealing hypoechoic, poorly defined areas. In selected cases, thyroid scintigraphy was performed to differentiate SAT from other causes of thyrotoxicosis and demonstrated low or absent radionuclide uptake, as expected in SAT.

Patients were excluded if they had an atypical or unclear clinical presentation, if they were diagnosed with an alternative cause of thyrotoxicosis (such as Graves’ disease, painless thyroiditis, or toxic nodular disease), or if they had recently received medications known to interfere with thyroid function, including amiodarone, iodinated contrast agents, lithium, corticosteroids, or antiepileptic drugs. Additional exclusion criteria included ongoing LT4 therapy at the time of SAT diagnosis and lack of adequate follow-up data to confirm the clinical course.

After applying the above-mentioned inclusion and exclusion criteria, we enrolled, under informed consent, 143 patients. Of these, 4 patients had continuously relapsing–recurrent SAT and were excluded from statistical analysis, which thus included 139 patients. Due to occasional missing data points for specific variables, the number of patients included in individual analyses may vary slightly.

For each patient, we considered the following parameters:

- Clinical parameters: age at diagnosis, Body Mass Index (BMI), smoking history, family history of thyroid disorders, maximum body temperature (afebrile if <37 °C, low-grade fever if between 37 °C and 38 °C, fever if >38 °C), pain in the thyroid lodge (0 if absent, 1 if mild, 2 if moderate and 3 if severe), month of onset and mean heart rate (HR).
- Biochemical parameters: TSH, FT4, FT3, thyroglobulin, erythrocyte sedimentation rate (ESR), C reactive protein (CRP), white blood cell count (WBC), anti-thyroglobulin (anti-Tg) autoantibodies (Abs), anti-thyroperoxidase (Anti-TPO) Abs.
- Instrumental parameters: decreased, increased, or normal tracer uptake at thyroid scintigraphy (if available), US parameters (thyroid size, presence of hypoechogenic areas, vascularization).

In line with the well-documented multiphasic course of subacute thyroiditis (SAT), the clinical progression of the disease was evaluated through the monitoring of TSH, FT4, and FT3 at three distinct timepoints, defined as TP: TP0 at the time of diagnosis; TP1, 4 to 8 weeks after diagnosis; TP2, 10 to 20 weeks after diagnosis; TP3, 28 to 54 weeks after diagnosis. When available, we included the thyroid US evaluation performed at TP3.

The US evaluation was conducted by expert Endocrinologists through high-quality machines, using linear US probes (frequency 5–11 MHz).

Patients were stratified at diagnosis based on thyrotoxicosis severity according to the following criteria:

(FT4 within the laboratory’s physiological reference range: 8.5–16.5 pg/mL)

- Mild thyrotoxicosis: FT4 1–1.5 times the upper limit of normal.
- Moderate thyrotoxicosis: FT4 1.5–2 times the upper limit of normal.
- Severe thyrotoxicosis: FT4 2–3 times the upper limit of normal.

Moreover, patients were divided into the “euthyroid”–patients who had regained full thyroid function–and the “hypothyroid” group–patients diagnosed with long-term hypothyroidism–according to their thyroid functional status at the last follow-up. “Long-term hypothyroidism” was defined as a persistent state of hypothyroidism (TSH levels > 4 μUI/mL) lasting 6.5 months or more after diagnosis (TP3). For patients who received LT4 treatment (see “treatment protocols below”):

- they were included in the “hypothyroid group” if they experienced a new rise in TSH levels (>4 μUI/mL) following LT4 reduction or withdrawal at the last follow-up;
- they were included in the “euthyroid group” if TSH levels following LT4 reduction or withdrawal were ≤4 μUI/mL at the last follow-up.

### 4.2. Treatment Protocols

Most patients were treated with NSAIDs per os, corticosteroids (prednisone, mean cumulative dose 1000 mg) per os, or both, based on their clinical features, preferring the use of corticosteroids in cases of moderate-to-severe symptoms. In cases of persistent symptoms, NSAIDs were administered in combination with corticosteroids. NSAIDs alone were employed in a minority of patients who presented with mild symptoms. The NSAID used was ibuprofen (1200 to 3200 mg daily divided into three doses for about 8 weeks).

If indicated, patients were started on levothyroxine (LT4) therapy at a dose of 1–1.3 mcg/kg/day between TP2 and TP3. Hypothyroidism was defined as a TSH level >4 μUI/mL. In our cohort, LT4 replacement therapy was initiated in patients with TSH levels above 10 μUI/mL, even if asymptomatic, or in those with TSH levels between 4 and 10 μUI/mL who exhibited symptoms of hypothyroidism. During follow-up, if recommended, LT4 was progressively reduced or discontinued based on TSH levels and clinical features.

### 4.3. Statistical Analysis

Outlier data points were identified through quantitative techniques, including the Z-score method and qualitative assessments. Regarding inferential statistics, group means were compared using the *t*-test under the assumption of normally distributed data. When the normality assumption was not met, the Mann-Whitney U test was applied as a nonparametric alternative. The chi-square test was employed to evaluate significant differences in the distributions of categorical variables, except for patient stratification and disease outcome analysis, where the Mann-Whitney U test was applied to ordinal variables. In the analysis of antibody titers and thyroid dimensions, one of the chi-square test’s assumptions was violated due to an expected frequency <5 in the contingency table. Thus, the reduced thyroid dimension group, which included only one patient, was excluded from the *p*-value calculation to ensure statistical validity. A *p*-value < 0.05 was considered statistically significant.

Of note, at TP3, FT4 and FT3 values in patients receiving LT4 therapy were approximated to zero to reflect the absence of sufficient endogenous thyroid hormone production. Consequently, FT4 and FT3 results at TP3 should be interpreted with caution.

Thyrotoxicosis stratification was incorporated into the Kaplan-Meier survival analysis, and the log-rank test was performed to assess differences in distributions and the association between thyrotoxicosis severity and time to hypothyroidism onset. Additionally, a Cox proportional hazards (Cox PH) model was used to estimate hazard ratios (HRs) and evaluate the impact of thyrotoxicosis severity on the risk of developing hypothyroidism over time.

Multivariable analysis was conducted using logistic regression to assess the relationship between disease outcomes and the instrumental, clinical, and biochemical parameters under study. Multicollinearity among the predictors was assessed using the Variance Inflation Factor (VIF) and Tolerance scores, ensuring that all values remained within acceptable limits (VIF < 10 and Tolerance > 0.1) to guarantee the stability and reliability of the regression coefficients.

## 5. Conclusions

Elevated FT3 and FT4 levels at diagnosis and specific TSH variations during follow-up emerged as strong indicators of disease progression, with ΔTSH (TP2-TP1) identified as an independent predictor at multivariable analysis. Severe thyrotoxicosis at diagnosis is associated with a faster progression to hypothyroidism, highlighting the need for closer monitoring in these patients, while the presence of anti-Tg antibodies, reduced thyroid volume, and persistent hypoechoic areas on ultrasound provide additional clinical utility as predictors of long-term hypothyroidism, offering deeper insights into the pathophysiology of SAT. Interestingly, traditional inflammatory markers were not associated with disease progression, suggesting that the mechanisms underlying SAT differ from those of classical autoimmune thyroiditis.

While treatment with glucocorticoids or NSAIDs did not significantly influence hypothyroidism risk in our study, the potential impact of disease severity and individual treatment responses warrants further investigation.

Despite the limitations of our retrospective study, our findings provide valuable tools for personalized patient care, allowing targeted follow-up and tailored interventions for those at higher risk of developing hypothyroidism. Future prospective studies are needed to validate our results and further refine predictive indicators for SAT outcomes.

## Figures and Tables

**Table 1:** Descriptive and inferential statistics of thyrotoxicosis stratification at disease presentation. Abbreviations: FT4, free thyroxine.

Thyrotoxicosis Severity	Euthyroid	Hypothyroid		Grand Total
Normal FT4	75.38% (49)	24.62% (16)		100% (65)
Mild	66.67% (24)	33.33% (12)		100% (36)
Moderate	42.11% (8)	57.89% (11)		100% (19)
Severe	41.67% (5)	58.33% (7)		100% (12)
Grand Total	65.15% (86)	34.85% (46)		100% (132)
*p*-value			**0.003**	

**Table 2:** Descriptive and inferential statistics of antibody positivity at disease presentation. Abbreviations: Anti-Tg, anti-thyroglobulin antibodies; Anti-TPO, anti-thyroid peroxidase antibodies.

Abs at TP0				
**Anti-Tg**	**Euthyroid**	**Hypothyroid**		**Grand Total**
Negative	70.80% (80)	29.20% (33)		100% (113)
Positive	42.31% (11)	57.69% (15)		100% (26)
Grand Total	65.47% (91)	34.53% (48)		100% (139)
*p*-value			**0.0059**	
**Anti-TPO**	**Euthyroid**	**Hypothyroid**		**Grand Total**
Negative	66.13% (82)	33.87% (42)		100% (124)
Positive	60.00% (9)	40.00% (6)		100% (15)
Grand Total	65.47% (91)	34.53% (48)		100% (139)
*p*-value			**0.64**	
**Combined**	**Euthyroid**	**Hypothyroid**		**Grand Total**
Negative	70.59% (72)	29.41% (30)		100% (102)
Positive	51.35% (19)	51.35% (18)		100% (37)
Grand Total	65.47% (91)	34.53% (48)		100% (139)
*p*-value			**0.035**	

**Table 3:** Descriptive and inferential statistics of antibody positivity against thyroid dimensions (diminished, increased, and normal) at disease presentation. Abbreviations: Anti-Tg, anti-thyroglobulin; Anti-TPO, anti-thyroid peroxidase antibodies.

**Anti-Tg**	**Diminished**	**Increased**	**Normal**		**Grand Total**
Negative	0.91% (1)	54.55% (60)	44.55% (49)		100% (110)
Positive	0.00% (0)	61.54% (16)	38.46% (10)		100% (26)
Grand Total	0.74% (1)	55.88% (76)	43.38% (59)		100% (136)
*p*-value				**0.55**	
**Anti-TPO**	**Diminished**	**Increased**	**Normal**		**Grand Total**
Negative	0.00% (0)	54.10% (66)	45.90% (56)		100% (122)
Positive	7.14% (1)	71.43% (10)	21.43% (3)		100% (14)
Grand Total	0.74% (1)	55.88% (76)	43.38% (59)		100% (136)
*p*-value				**0.12**	
**Combined**	**Diminished**	**Increased**	**Normal**		**Grand Total**
Negative	0.00% (0)	54.00% (54)	46.00% (46)		100% (100)
Positive	2.78% (1)	61.11% (22)	36.11% (13)		100% (36)
Grand Total	0.74% (1)	55.88% (76)	43.38% (59)		100% (136)
*p*-value				**0.63**	

**Table 4:** Descriptive and inferential statistics of US findings at disease presentation. Abbreviations: TP0, time point 0 (disease presentation); US, ultrasound.

US findings at TP0				
**Dimensions**	**Euthyroid**	**Hypothyroid**		**Grand Total**
Diminished	100% (1)	0% (0)		100% (1)
Increased	69.74% (53)	30.26% (23)		100% (76)
Normal	59.32% (35)	40.68% (24)		100% (59)
Grand Total	65.44% (89)	34.56% (47)		100% (136)
*p*-value			**0.21**	
**Hypoechoic areas**	**Euthyroid**	**Hypothyroid**		**Grand Total**
Absent	71.43% (5)	28.57% (2)		100% (7)
Single	75.00% (3)	25.00% (1)		100% (4)
Multiple, monolateral	66.66% (10)	33.33% (5)		100% (15)
Multiple, bilateral	65.18% (73)	34.82% (39)		100% (112)
Grand Total	65.94% (91)	34.06% (47)		100% (138)
*p*-value			**0.98**	
**Nodules**	**Euthyroid**	**Hypothyroid**		**Grand Total**
Absent	69.49% (41)	30.51% (18)		100% (59)
Present	66.66% (34)	33.33% (17)		100% (51)
Pseudo-nodular	57.14% (16)	48.86% (12)		100% (28)
Grand Total	65.94% (91)	34.06% (47)		100% (138)
*p*-value			**0.52**	
**Vascularization**	**Euthyroid**	**Hypothyroid**		**Grand Total**
Increased	59.26% (16)	40.74% (11)		100% (27)
Normal	73.03% (65)	26.97% (24)		100% (89)
Reduced	45.45% (10)	54.55% (12)		100% (22)
Grand Total	65.94% (91)	34.06% (47)		100% (138)
*p*-value			**0.036**	

**Table 5:** Descriptive and inferential statistics of US characteristics at TP3. Abbreviations: TP3, time point 3 (last follow-up); US, ultrasound.

US findings at TP3				
**Dimensions**	**Euthyroid**	**Hypothyroid**		**Grand Total**
Diminished	33.33% (6)	66.66% (12)		100% (18)
Increased	100% (2)	0.00% (0)		100% (2)
Normal	69.83% (81)	30.17% (35)		100% (116)
Grand Total	65.44% (89)	34.56% (47)		100% (136)
*p*-value			**0.003**	
**Hypoechoic areas**	**Euthyroid**	**Hypothyroid**		**Grand Total**
Absent	79.49% (31)	20.51% (8)		100% (39)
Present	59.60% (59)	40.40% (40)		100% (99)
Grand Total	65.22% (90)	34.78% (48)		100% (138)
*p*-value			**0.027**	
**Nodules**	**Euthyroid**	**Hypothyroid**		**Grand Total**
Absent	62.90% (39)	37.10% (23)		100% (62)
Present	65.45% (36)	34.55% (19)		100% (55)
Pseudo-nodular	71.43% (15)	28.57% (6)		100% (21)
Grand Total	65.22% (90)	34.78% (48)		100% (138)
*p*-value			**0.297**	
**Vascularization**	**Euthyroid**	**Hypothyroid**		**Grand Total**
Increased	80.00% (8)	20.00% (2)		100% (10)
Normal	63.49% (80)	36.51% (46)		100% (126)
Reduced	100% (2)	0.00% (0)		100% (2)
Grand Total	65.22% (90)	34.78% (48)		100% (138)
*p*-value			**0.334**	
**Resolution**	**Euthyroid**	**Hypothyroid**		**Grand Total**
Not achieved	59.60% (59)	40.40% (40)		100% (99)
Achieved	84.38% (27)	15.63% (5)		100% (32)
Grand Total	65.65% (86)	34.35% (45)		100% (131)
*p*-value			**0.01**	

**Table 6:** Multivariable analysis results. The constant reflects the model’s predicted odds of long-term hypothyroidism when all predicted variables are set to zero; the low odds ratio is expected given that the condition is relatively uncommon. Abbreviations: B, regression coefficient; SE, standard error; z, z-score; *p*, *p*-value; US, ultrasound; ΔTSH, change in thyroid-stimulating hormone; TP1, time point 1; TP2, time point 2; OR, odds ratio; CI, confidence interval; Anti-Tg Abs, anti-thyroglobulin antibodies.

	Coefficient B	Standard Error	z	*p*	Odds Ratio	95% Conf. Interval
**Constant**	−1.9	0.59	3.23	**0.001**	0.15	0.05–0.47
**Anti-Tg Abs positivity**	2.23	1.14	1.95	**0.051**	9.33	0.99–87.95
**US—reduced vascularization**	1.08	0.96	1.12	**0.262**	2.93	0.45–19.22
**US—increased vascularization**	−0.08	0.89	0.08	**0.932**	0.93	0.16–5.35
**Δ TSH (TP2-TP1)**	0.26	0.1	2.71	**0.007**	1.3	1.07–1.57
**US—reduced dimensions**	1.83	1.12	1.64	**0.102**	6.21	0.7–55.43
**US—increased dimensions**	−19.02	7380.73	0	**0.998**	0	0–infinity

## Data Availability

The data presented in this study are available on request from the corresponding author.

## Supplementary Materials

The following supporting information can be downloaded at: https://www.mdpi.com/article/10.3390/ijms26094176/s1.
