# Supplementary material for: Biochemical and Ultrasonographic Parameters Predicting Long-Term Hypothyroidism After Subacute Thyroiditis
Source: Int J Mol Sci. 2025 Apr 28;26(9):4176. doi: 10.3390/ijms26094176 (PMC12071259; doi:10.3390/ijms26094176)
Supplement: Supplementary file 1 [file ijms-26-04176-s001.zip › ijms-3534706-supplementary.pdf]

Supplementary Table S1. *Descriptive and inferential statistics of parameters at disease presentation.*

**TP0**			
**Age**	**Euthyroid**		**Hypothyroid**
Mean	49.07		47.65
Median	48.00		48.00
St. Deviation	10.39		10.16
p-value		**0.44**	
**BMI**	**Euthyroid**		**Hypothyroid**
Mean	24.00		23.96
Median	23.40		23.98
St. Deviation	3.55		3.14
p-value		**0.95**	
**Gender**	**Euthyroid**		**Hypothyroid**
Females number	75		36
Males number	15		12
Total	91		48
p-value		**0.52**	
**Habits**	**Euthyroid**		**Hypothyroid**
Non-smokers	82		45
Smokers	7		1
Ex-smokers	2		2
p-value		**0.094**	
**Season**	**Euthyroid**		**Hypothyroid**
First trimester	17		11
Second trimester	22		10
Third trimester	24		15
Fourth trimester	28		12
p-value		**0.29**	
**Pain**	**Euthyroid**		**Hypothyroid**
No pain	50% (1)		50% (1)
Mild pain	75% (18)		25% (6)
Moderate pain	64.44% (58)		35.56% (32)
Severe pain	60.87% (14)		39.13% (9)
p-value		**0.094**	
**Max temperature**	**Euthyroid**		**Hypothyroid**
Afebrile	65.67% (44)		34.33% (23)
Low-grade T°	76.67% (23)		23.33% (7)
Fever	57.14% (24)		42.86% (18)
p-value		**0.23**	
**Heart rate**	**Euthyroid**		**Hypothyroid**
Mean	82.64		82.21
Median	80		78
St. Deviation	10.37		11.89
p-value		**0.53**	
**ESR (mm/h)**	**Euthyroid**		**Hypothyroid**
Mean	52.39		53.49
Median	52.39		53.00
St. Deviation	27.32		27.32
p-value		**0.41**	0.41
**CRP (mg/L)**	**Euthyroid**		**Hypothyroid**
Mean	24.37		24.12
Median	10.59		13.60
St. Deviation	34.56		27.72
p-value		**0.47**	
**WBC (x10^9/L)**	**Euthyroid**		**Hypothyroid**
Mean	9.49		9.03
Median	9.62		8.70
St. Deviation	2.38		2.27
p-value		**0.15**	

Supplementary Table S2. *Descriptive and inferential statistics of treatment parameters.*

Treatment			
**Use**	**Euthyroid**		**Hypothyroid**
NSAIDs	50% (9)		50% (9)
Glucocorticoids	65.52% (38)		34.48% (20)
Both	73.08% (38)		26.92% (14)
No medication	54.55% (6)		45.45% (5)
**NSAIDs**	**Euthyroid**		**Hypothyroid**
No NSAIDs	63.77% (44)		36.23% (25)
NSAIDs	67.14% (47)		32.86% (23)
Total	65.47% (91)		34.53% (48)
p-value		**0.6**	
**Glucocorticoids**	**Euthyroid**		**Hypothyroid**
Glucocorticoids	69.09% (76)		30.91% (34)
No Glucocorticoids	51.72% (15)		48.28% (14)
Total	65.47% (91)		34.53% (48)
p-value		**0.08**	

Supplementary Table S3. *Descriptive and inferential statistics ΔTSH at different time points.*

Change in TSH			
**ΔTSH (TP1-TP0)**	**Euthyroid**		**Hypothyroid**
Mean	1.58		4.08
Median	1.10		0.29
St. Deviation	2.00		7.20
p-value		**0.3**	
**ΔTSH (TP2-TP0)**	**Euthyroid**		**Hypothyroid**
Mean	1.71		8.12
Median	1.49		5.21
St. Deviation	1.72		8.90
p-value		**<0.001**	
**ΔTSH (TP3-TP1)**	**Euthyroid**		**Hypothyroid**
Mean	1.57		6.13
Median	1.50		5.59
St. Deviation	0.97		2.86
p-value		**<0.001**	
**ΔTSH (TP2-TP1)**	**Euthyroid**		**Hypothyroid**
Mean	0.15		8.69
Median	0.00		6.20
St. Deviation	2.85		10.42
p-value		**<0.001**	
**ΔTSH (TP3-TP1)**	**Euthyroid**		**Hypothyroid**
Mean	-0.02		4.54
Median	0.39		5.65
St. Deviation	2.04		5.24
p-value		**0.005**	
**ΔTSH (TP3-TP2)**	**Euthyroid**		**Hypothyroid**
Mean	-0.11		3.15
Median	-0.02		3.18
St. Deviation	1.50		2.99
p-value		**<0.001**	

Supplementary Table S4. *Descriptive and inferential statistics of LT4 supplementation*

**Levothyroxine at TP3**			
**LT4**	**Euthyroid**		**Hypothyroid**
Not Necessary	81.25% (91)		18.75% (21)
Necessary	0.00% (0)		100% (27)
Total	65.47% (91)		34.53% (48)
p-value		**<0.001**	

Supplementary Table S5. *Variable selection for multivariate analysis.*

Variable	Univariate Analysis p-value	Inclusion	Rationale for Exclusion
**TSH TP0**	**0.04**	**X**	Multicollinearity with more relevant parameters
**FT4 TP0**	**0.01**	**X**	Multicollinearity with more relevant parameters
**FT3 TP0**	**0.01**	**X**	Low clinical relevance when compared to others
**Anti Tg**	**0.0059**	**V**	None
**US Vascularization**	**0.036**	**V**	None
**TSH TP2**	**0.001**	**X**	Multicollinearity with more relevant parameters
**TSH TP3**	**0.001**	**X**	Multicollinearity with more relevant parameters
**FT4 TP2**	**0.01**	**X**	Multicollinearity with more relevant parameters
**FT4 TP3**	**0.001**	**X**	Multicollinearity with more relevant parameters
**FT3 TP3**	**0.001**	**X**	Multicollinearity with more relevant parameters
**Δ TSH (TP2-TP0)**	**0.001**	**V**	None
**Δ TSH (TP3-TP0)**	**0.001**	**X**	Unreliable due to LT4 utilization
**Δ TSH (TP2-TP1)**	**0.001**	**V**	None
**Δ TSH (TP3-TP1)**	**0.005**	**X**	Unreliable due to LT4 utilization
**Δ TSH (TP3-TP2)**	**0.001**	**X**	Unreliable due to LT4 utilization
**LT4**	**0.001**	**X**	Multicollinearity with more relevant parameters
**US Dimensions**	**0.003**	**V**	None

Supplementary Figure S1. *The Boxplot displays the TSH distribution across different timepoints categorized into disease progression groups. The accompanying table provides descriptive statistics for each group and timepoint.*

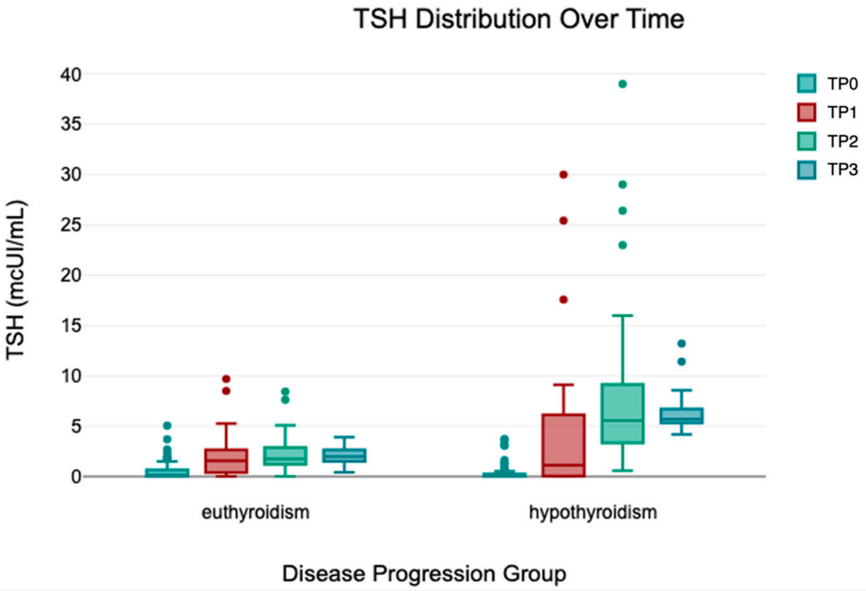

		TSH (mcUI/mL)												
Time Points	Disease Progression	Mean	Median	Mode	Std. Deviation	Minimum	Maximum	Range	Quartile 1	Quartile 2	Quartile 3	Interquartile Range	Skew	Kurtosis
TP0	euthyroidism	0.49	0.08	0.01	0.84	0.01	5.05	5.04	0.02	0.08	0.66	0.64	2.96	10.95
	hypothyroidism	0.46	0.03	0.01	0.97	0.01	3.74	3.73	0.01	0.03	0.26	0.25	2.48	5.19
TP1	euthyroidism	1.67	1.57	0.01	1.95	0.01	9.68	9.67	0.37	1.57	2.65	2.28	1.86	4.82
	hypothyroidism	4.29	1.11	0.01	7.16	0.01	30	29.99	0.03	1.11	6.13	6.1	2.39	5.85
TP2	euthyroidism	1.97	1.74	1.5	1.6	0.02	8.45	8.43	1.2	1.74	2.9	1.7	1.72	4.47
	hypothyroidism	8.61	5.58	3.9	8.79	0.6	39	38.4	3.33	5.58	9.15	5.82	2.01	3.94
TP3	euthyroidism	2.08	1.96	2.3	0.8	0.43	3.9	3.47	1.49	1.96	2.65	1.16	0.26	-0.64
	hypothyroidism	6.24	5.71	5.71	2.28	4.18	13.2	9.02	5.31	5.71	6.73	1.41	1.86	3.21
